# Editorial: Methodologies to improve the performance of chondrocytes for cartilage repair and regeneration

**DOI:** 10.3389/fbioe.2023.1335134

**Published:** 2023-11-22

**Authors:** Gina Lisignoli, Giovanna Nalesso, Andrea Barbero

**Affiliations:** ^1^ Laboratorio di Immunoreumatologia e Rigenerazione Tissutale, IRCCS Istituto Ortopedico Rizzoli Bologna, Bologna, Italy; ^2^ Department of Comparative Biomedical Sciences, School of Veterinary Medicine, University of Surrey, Guildford, United Kingdom; ^3^ Cartilage Engineering, Department of Biomedicine, University Hospital and University of Basel, Basel, Switzerland

**Keywords:** chondrocytes, cartilage repair, osteoarthritis, tissue engineering, mesenchymal stromal cells (MSC)

The articular cartilage has a limited capacity for self-repair. Consequently, traumatic cartilage defects, if not properly treated, may develop in osteoarthritis (OA) (Punzi et al., 2016), a degenerative joint disease that causes pain, reduction of mobility and quality of life of hundreds of million people worldwide (Abramoff and Caldera, 2020). Transplantation of autologous *ex vivo* cultured/manipulated cells exhibiting pro-chondrogenic and/or anti-inflammatory effects is an effective method to treat cartilage defects and prevent their further degeneration (Tang and Wang, 2015).

Autologous articular chondrocytes (AC) implantation (ACI) was the first cell-based therapy that used a tissue engineering process to repair cartilage defects (Brittberg et al., 1994). Such procedure require the isolation of these therapeutic cells from a small biopsy, their expansion *in vitro*, and their transplantation in the defect, directly, or after being cultured onto biodegradable scaffolds. Although medium- and long-term results regarding this procedures are satisfactory, still a certain percentage of autologous AC-based procedures lead to unsuccessful and unsatisfactory clinical outcomes for the treatment of focal cartilage lesions (Pestka et al., 2014), probably due to the unpredictable acquisition of morphological alterations by these cells during their *ex vivo* culture. Mesenchymal stromal cells (MSC) that can be isolated from different adult tissue like bone marrow and fat have been proposed as alternative source of cartilage-regenerating cells, especially for their immune modulatory properties (Pers et al., 2018). Yet, their performance has been inconsistent across donors/studies (Mamidi et al., 2016). Hence there is a strong need to develop new methodologies to improve the performance of transplanted cells and to identify variables which can predict a successful healing process.

In this Research Topic, multiple authors contributed to improve our understanding on these subjects. Frerker et al. demonstrated the best performance of AC than MSC free-scaffold disc for tissue engineering of articular cartilage. They evidenced by proteomics analysis more hypertrophic proteins in MSC than in AC and by sequencing analysis more microRNAs associated with normal cartilage in AC than in MSC.

Jing et al. unveiled the therapeutic effects of Flavokawain A (FKA), a plant extract, on chondrocytes. Intriguingly, the authors demonstrated *in vitro* that FKA inhibits IL1β-induced inflammatory factors, regulates autophagy and senescence-related genes and induced the chondrogenic markers. Moreover, they demonstrated in a murine model of OA a promising impact of FKA on cartilage degradation and subchondral bone remodelling.

Finally, Vignon et al., provided preliminary evidences that blood derived CD34^+^ cells could also exert some anti-OA effects *in vitro* and in small animal model (Vignon et al.). Follow up studies in large osteoarthritic OA animal model, like the one used recently by Acevedo Rua et al. (2021) would then be required to consolidate the therapeutic efficacy of these cells.

As summarized by Herberts et al. (2011), (stem) cell therapy is associated with several risk factors including the source of cells, their proliferation capacity, *in vitro* culture and/or other manipulation steps. To overcome these limitations, several scientists have investigated the therapeutic potential of the secretome of different cell types including AC and MSC (Velot et al., 2021). A few studies using different animal models have shown the capacity of specific extracellular vesicles types to decrease OA symptoms *in vivo* (Cosenza et al., 2017; Zhu et al., 2017). However, protocols to generate and store this bioactive mixture must be optimized and standardized before components of the secretome could be used in the clinic.

In this Research Topic, (Jammes et al.) evidenced that conditioned media (CM) stored at different temperature and for different times as well as the use different cytokines during the cell (MSC and AC) conditioning can modulate the anti-inflammatory and pro-anabolic activities of the CM. The authors further suggest that MSC secretome can be used during the amplification of AC *in vitro* to counteract their tendency to acquire aberrant phenotypic changes associated with cell expansion, ultimately enhancing their regenerative potential in ACI procedures.

In the context of the present research, it is important to highlight the key role of the biomechanical microenvironment to modulate cell regenerative properties including proliferation, matrix synthesis and secretion of bioactive factors. Considering that mechanobiology and chronobiological signaling pathways are closely interconnected during cartilage formation and maintenance, the combination of biochemical stimulation and chronobiology might represent an innovative approach to augment the performance of chondrocytes or other cell types for cartilage repair/regeneration (Vago et al.).

We believe that the methodologies and approaches investigated in this Research Topic are interesting starting points and considerations to be reflected upon for the development of novel or the improvement of existing cell-based approaches to cartilage repair. This Research Topic highlights that among the remaining challenges that still need to be resolved, «the standardization of processing and manufacture to obtain a consistent product of defined identity and known potency to patient benefit» (Zelinka et al., 2022) is crucial for the successful clinical implementation of novel cell-based regenerative therapeutics.
